# Modeling study of divertor particle flow pattern and in–out plasma density asymmetry due to drifts with SOLPS and BOUT++

**DOI:** 10.1038/s41598-023-36696-y

**Published:** 2023-06-12

**Authors:** Guozhong Deng, Changhui Yu, Xiaomei Wang, Wei Feng, Xiaoju Liu, Haihua Yang

**Affiliations:** 1 Key Laboratory of Radiation Oncology of Taizhou, Radiation Oncology Institute of Enze Medical Health Academy, Department of Radiation Oncology, Taizhou Hospital Affiliated to Wenzhou Medical University, Taizhou, 317000 Zhejiang Province China; 2 Institute of Plasma Physics, Chinese Academy of Sciences, Hefei, 230031 China

**Keywords:** Plasma physics, Magnetically confined plasmas

## Abstract

A study of the effects of drifts on the particle flow pattern and in–out divertor plasma density asymmetry for L-mode and H-mode plasmas is carried out for EAST discharges by the edge plasma transport codes SOLPS and BOUT++ . The simulation of L-mode plasmas is done by SOLPS while the simulation of H-mode plasmas is done by BOUT++ . The toroidal magnetic field direction for the simulated discharge is artificially reversed in the codes to study the effects of different drift directions on the divertor particle flow pattern and the in–out asymmetry of divertor plasma density. The divertor particle flows induced by diamagnetic and E × B drifts are found to have similar directions in the divertor region for the same discharge. The directions of the flows induced by drifts would be reversed with the reverse of toroidal magnetic field direction. The diamagnetic drift seems to have no effect on the in–out asymmetry of divertor plasma density due to its divergence-free nature. However, the E × B drift could result in a pronounced asymmetry of plasma density between the inner and outer divertor targets. The density in–out asymmetry caused by E × B drift is reversed with the reverse of E × B drift flow direction. Detailed analysis shows that the radial component of the E × B drift flow is the main cause of density asymmetry. The results from the simulation of H-mode plasmas with BOUT++ are similar to those of the L-mode plasmas with SOLPS except that the drift effects seem to be slightly larger in the H-mode plasmas compared to the L-mode plasmas.

## Introduction

The divertor is the most important component of a tokamak device for the exhaust of particles and fusion power^1^. The distributions of plasma density and particle flow in the divertor region are essential to the edge particle and energy balance. The particles from the core plasmas along the magnetic field lines in the scrape-off layer (SOL) to the divertor, are usually found to be asymmetrically distributed between the inner and outer divertor targets^2^. Understanding the in–out asymmetry of divertor plasma density is of great importance to the design and operation of future high-power and long-pulse tokamaks like ITER and China Fusion Engineering Test Reactor (CFETR).

The experimental study of divertor in–out asymmetry of plasma density has been carried out worldwide on present tokamaks such as DIII-D^3–5^, JET^6–8^, ASDEX-Upgrade^9–11^ and JT-60U^12–14^. The basic finding is the reversed asymmetric behavior with the reverse of toroidal magnetic field. The density at the inner divertor target is usually larger than that of the outer divertor target for lower single null (LSN) discharges with normal toroidal magnetic field (ion B × ▽B direction towards the lower X-point), while the asymmetry is reversed with reversed toroidal magnetic field (ion B × ▽B direction directed away from the lower X-point). A common explanation to the strong asymmetry is due to the existence of various types of plasma drifts, including the diamagnetic and E × B drifts^15–19^. Presently, the E × B drift has been widely recognized as the most important factor in enhancing the in–out asymmetry. However, there is still dispute over which component (radial or poloidal) of the E × B drift plays the leading role. Rozhansky et al. presented that the poloidal E × B drift was the main factor in enhancing the in–out asymmetry of divertor plasma density flux based on the study of sheath boundary condition at targets^17,18^. However, Chankin found that the radial E × B drift was playing the dominant role by analyzing the convective flows caused by the poloidal and radial components of the E × B drift based on a series of EDGE2D-EIRENE modeling^19^. Although the diamagnetic drift is considered not to be important in inducing the asymmetry due to its divergence-free nature^20–22^, its actual effect on the in–out asymmetry of divertor plasma density has not been widely discussed yet.

EAST is China’s first superconducting Tokamak device with a divertor and configuration similar to ITER. Recently, the experimental studies have been conducted on the in–out asymmetry of divertor particle flux of EAST H-mode discharges^23,24^, showing that the asymmetry is reversed with the reverse of the toroidal magnetic field direction. SOLPS simulations also provide the asymmetric behaviors similar to the experiment^25–27^. However, these previous simulation researches mainly focus on the qualitative consistency between simulations and experiments, without enough efforts at the quantitative level. More researches are needed on the impact of different types of drift on the particle flow patterns in the divertor region, which is crucial to the formation of in–out divertor density asymmetry. Understanding these physics will have special significance for the high-power long pulse operation of EAST in the future, which is the main research focus of this article.

The rest of the paper is organized as follows. Section “Simulation setups” briefly introduces the simulation setups of the SOLPS and BOUT++ edge plasma simulation codes. The effects of different types of drifts on the particle flow pattern and in–out density asymmetry in the divertor region for the simulation of L-mode plasmas by SOLPS are discussed in Sect. “Effects of drifts on the particle flow pattern and in–out asymmetry of plasma density in the divertor region for L-mode simulations with SOLPS”. The corresponding results for the simulation of H-mode plasmas by BOUT++ are presented in Sect.  “Effects of drifts on the particle flow pattern and in–out asymmetry of plasma density in the divertor region for H-mode simulations with BOUT++ ” Finally, all the results are summarized in Sect. “Summary and conclusions”.

## Simulation setups

Two edge plasma transport codes are used in this work, i.e., the SOLPS code, as well as the BOUT++ transport code. SOLPS is a large edge plasma code package consisting of a plasma fluid code B2.5 and a kinetic Monte-Carlo neutral code Eirene^28^. The code has been extensively used for edge plasma simulations on EAST^25–30^. The BOUT++ is a framework for implementing 2D and 3D plasma/fluid simulation in curvilinear geometry^31,32^. Many physical models have been developed in this framework and the transport model is one of them^33–35^. Both codes solve the same set of fluid equations in the edge plasma region based on field line aligned coordinate systems. Although the forms of the fluid equations solved in BOUT++ and SOLPS are different, with BOUT++ solving the continuity, temperature and velocity equations and SOLPS solving the particle, energy and momentum conservation equations, the equations in both codes are derived from the Braginskii equations ^36^. The simulated distributions of plasma parameters like the plasma density and temperature in the whole mesh area are determined by the boundary conditions and the radial thermal and particle transport coefficients for both codes. The sources of plasmas are from the core–edge interface (i.e. the inner boundary) and the recycling at wall (i.e. the outer boundary) and the divertor targets. The recycling neutral is D^0^ and the recycling coefficient is 0.65 at wall and 1.0 at the divertor targets. The reason to the adoption of this set of recycling coefficients is due to the good match of the simulated density profile with the experiment in our previous work^33^. Neutral models are included in both codes and are applied in the simulations in this work. However, the forms of the neutral models in these two codes are different. The SOLPS has a kinetic Monte-Carlo neutral code Eirene^28^ while a fluid neutral model is implemented in BOUT++ ^33^. The sources of the neutrals for both codes are from the particle recycling at wall and divertor targets. The magnitudes of the neutral densities at the outer mid-plane and divertor surfaces are in the range of 1.0–15.0 (10^16^ m^−3^) while the corresponding values for the plasma densities are roughly in the range of 2.0–30.0 (10^18^ m^−3^). Note that direct comparisons of divertor target parameters from SOLPS and BOUT++ simulations along with the experiment have been made in our previous work with reasonable agreements being achieved^33,34^, which show that both of the two codes are reliable in the edge plasma simulations of EAST discharges.

**Figure 1:**
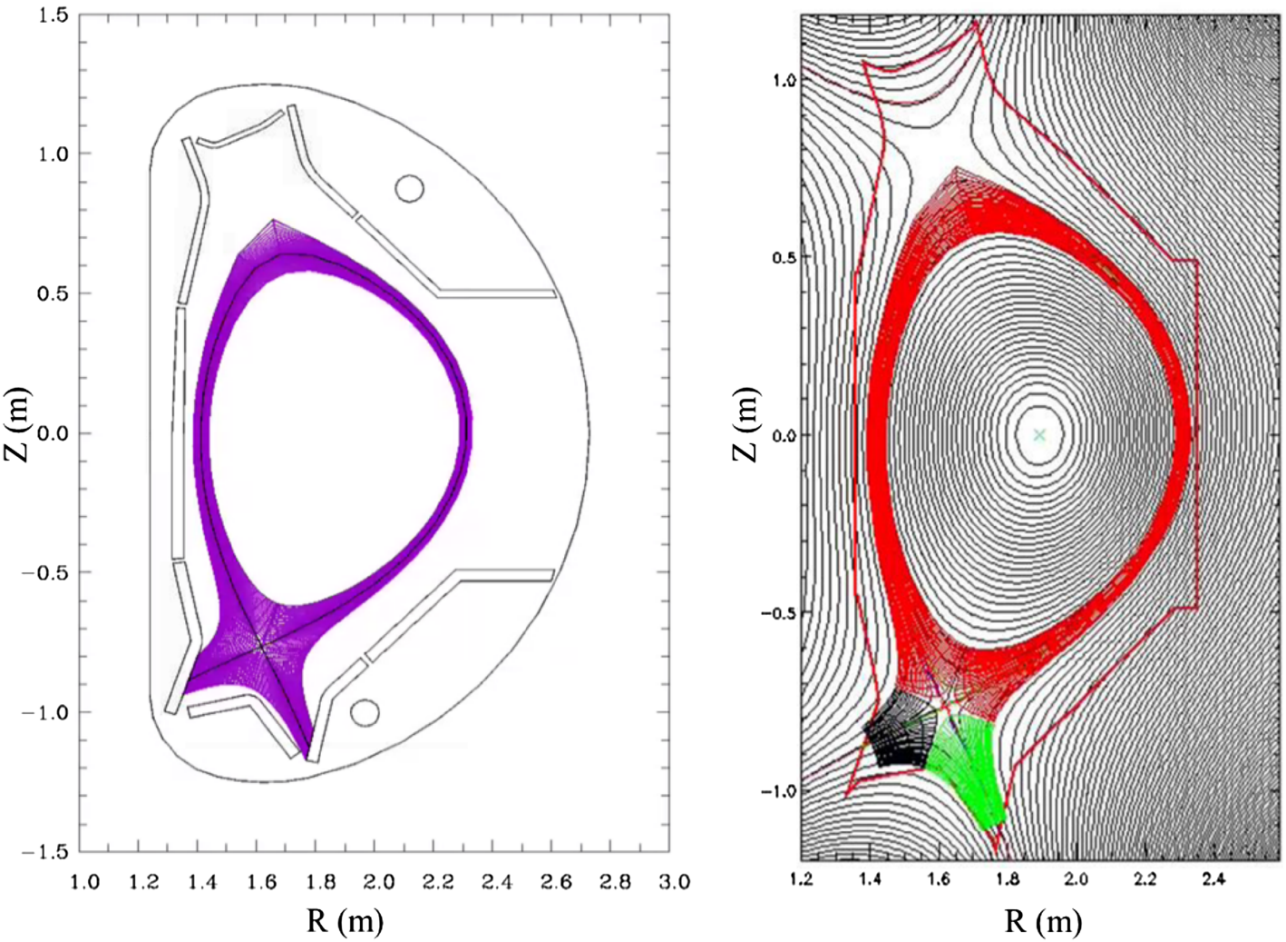
The grid images of EAST shot #48337 for SOLPS simulation (left) and BOUT++ simulation (right). The SOLPS grid is generated by the built-in code called CARRE^37^ while the BOUT++ grid is generated by the subpackage named HYPNOTOAD^31^.

**Figure 2:**
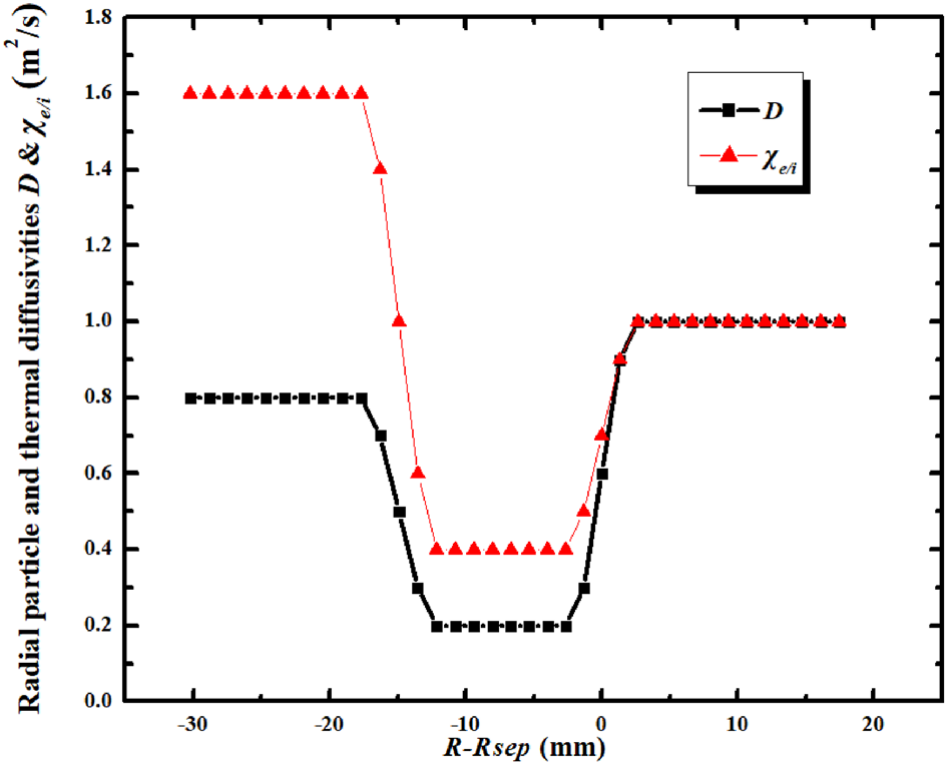
The radial plasma particle and thermal transport coefficient profiles for the BOUT++ simulations of EAST discharge #48337. *D*: the radial particle diffusivity, *χ*_*e/i*_: the radial energy diffusivity; *R*_sep_: the radial location of the separatrix, *R*: the radial coordinate of the mesh area.

**Figure 3:**
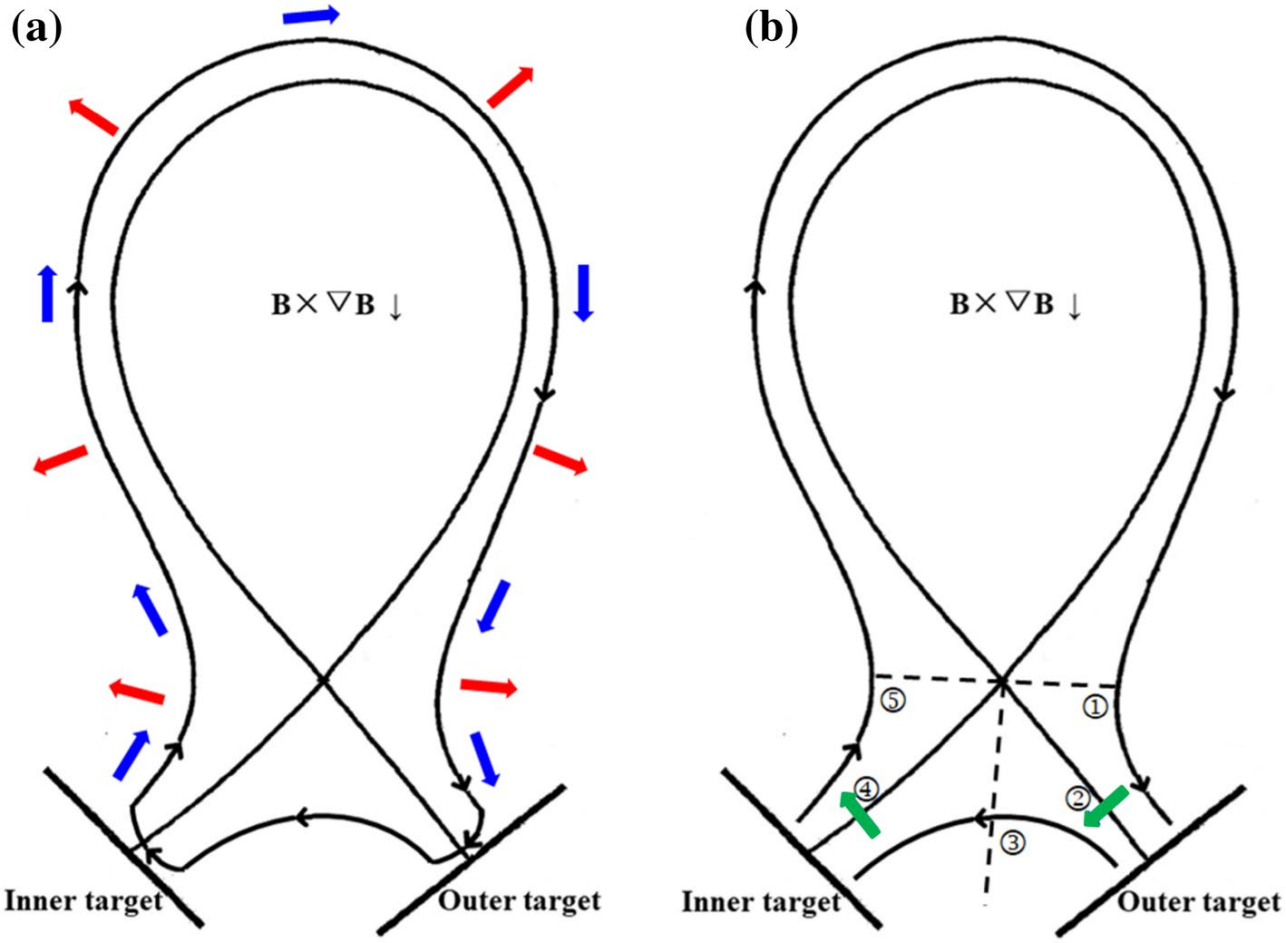
The diagrams of the directions of diamagnetic drift flow (**a**) and E × B drift flow (**b**) in the SOL and PFR regions for tokamak plasmas with normal toroidal magnetic field. In Fig. 3a, the black arrows represent the direction of diamagnetic drift flow, the blue arrows represent the poloidal direction and the red arrows represent the radial direction. In Fig. 3b, the black arrows represent the direction of poloidal E × B drift flow while the green arrows represent the direction of radial E × B drift flow. ①: interface of the low field side upstream and the outer divertor region; ②: interface of the SOL and PFR at the outer divertor region; ③: interface of the outer and inner PFR region; ④: interface of the SOL and PFR at the inner divertor region; ⑤: interface of the high field side upstream and the inner divertor region.

An EAST H-mode discharge #48337 is chosen for the simulation. It is a LSN discharge with ion B × ▽B direction towards the X-point. The plasma current and toroidal magnetic field for this discharge are 0.4 MA and 2.2 T respectively. The simulation grids are generated based on the magnetic equilibrium of this discharge from the kinetic EFIT^38^. Figure 1 shows the grid images for SOLPS and BOUT++ with the resolution of 36 × 96 and 36 × 64 respectively (radial × poloidal). Note that the different colors in the right figure of Fig. 1 have nothing to do with the simulation process. The BOUT++ code would set different colors for different regions when it generates the grid. The species included in the simulation are D^0^, D^+^ and electrons with no impurities from seeding or sputtering at divertor targets. For the SOLPS simulation, the density at core–edge interface is set to be 1.6 × 10^19^ m^−3^ and power into the simulation domain is set to be 0.8 MW. The radial transport coefficients for particles (*D*) and energy (*χ*_*e/i*_) are set to be 0.4 m^2^/s and 1.6 m^2^/s in the entire simulation domain, which are common practices for the EAST L-mode plasma simulations^30^. For the BOUT++ simulation, the density and temperature at CEI are set to be 2.8 × 10^19^ m^−3^ and 450 eV respectively. The “U” shaped profiles of transport coefficients are set in the simulation as shown in Fig. 2. The coefficients at the pedestal region are set to be small due to the existence of the transport barrier there, which are common practices in typical H-mode plasma simulations^26,29,30^. The ion thermal diffusivity is set to be the same as the electron thermal diffusivity for simplicity. *R*_sep_ shown in Fig. 2 is the radial location of the separatrix. The positive radial direction is shown in Fig. 3a, so the positive values of *R*-*R*_sep_ is the radial locations of the simulated area outside separatrix and the negative values of *R*-*R*_sep_ is the radial locations of the simulated area inside separatrix. Note that the transport coefficients are by default poloidally constant for the simulation.

For both the SOLPS and BOUT++ simulations, four cases are run, i.e., w/o drifts, w/ only diamagnetic drift, w/ only E × B drift and w/ all drifts. Another four cases are run based on the four cases above but with artificially reversed toroidal magnetic field to study the effects of different drift directions on the particle flow pattern and divertor asymmetry. Note that our initial plan was to simulate the H-mode plasmas with both SOLPS and BOUT++ and make direct comparison between the two. However, the SOLPS cases w/ drifts are hard to converge with “U” shaped profiles of transport coefficients. The L-mode simulations with SOLPS are carried out in this work.

## Effects of drifts on the particle flow pattern and in–out asymmetry of plasma density in the divertor region for L-mode simulations with SOLPS

As aforementioned, the L-mode cases with different drift scenarios and toroidal field directions are run by the SOLPS code. In this section we will discuss in detail on the effects of different drifts and their directions on the particle flow pattern and in–out asymmetry of plasma density in the divertor region for these L-mode plasmas.

As we all know, plasma drifts could lead to the change of particle flow pattern in the edge region Figure 3a,b show the diagrams of diamagnetic and E × B drift flow directions in the SOL and private flux region (PFR) for tokamak plasmas with LSN configuration under normal toroidal magnetic field. The black arrows represent the direction of poloidal E × B drift flows while the green arrows represent the direction of radial E × B drift flows in Fig. 3b. The directions of all the drift flows would be reversed with the reverse of toroidal magnetic field. 5 interfaces are marked in Fig. 3b with the descriptions in the caption.

Theoretically, the diamagnetic drift is considered to be divergence free and the flow would change direction near the outer and inner targets as shown in Fig. 3a, thus causing no net flows to the divertor surface^20–22^. The assumption made in this process is that the variation of the magnetic field along the radial direction could be neglected as it is a small term, i.e. ∂**B**/ ∂r = 0. The detailed derivation of the formulas for this physical process is extremely complex and can be found in^21^. The general concept of this theory is that the diamagnetic drift flow would change its direction before hitting the divertor target surface due to the parallel pressure gradient encountered at the magnetic pre-sheath^39^ in front of the target plate, which allows the diamagnetic drift direction to turn from a mostly poloidal to a radial heading. However, both the SOLPS and BOUT++ do not solve the magnetic pre-sheath region and the sheath boundary conditions are imposed at the entrance of the magnetic pre-sheath region for both codes, which indicate that the results from our simulations may differ from these mathematical evaluations to some extent.

**Figure 4:**
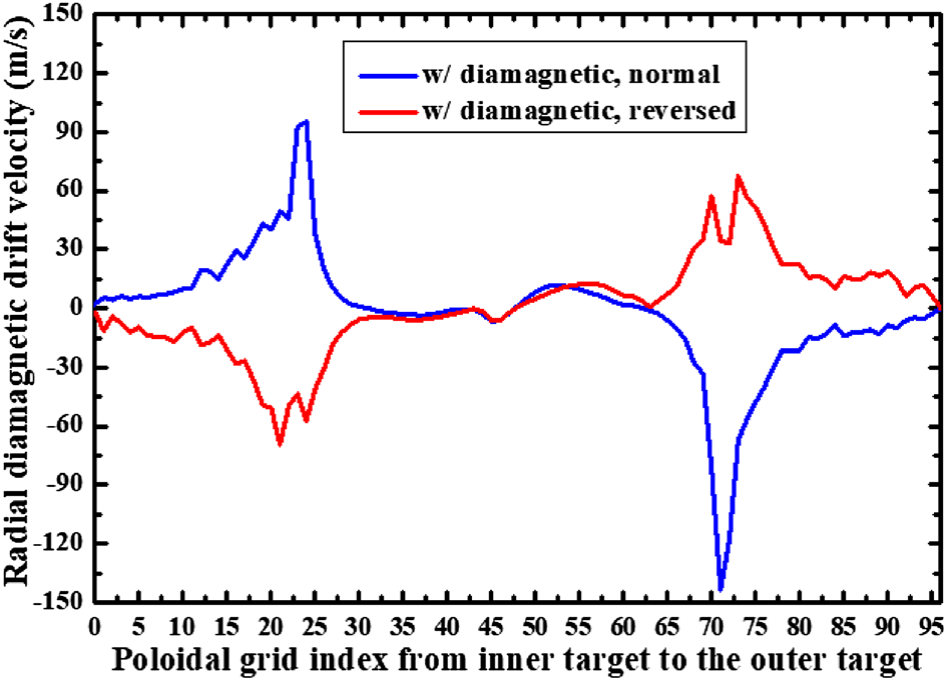
The radial diamagnetic drift velocities at separatrix for the simulation cases with only diamagnetic drift under normal and reversed toroidal field directions.

**Figure 5:**
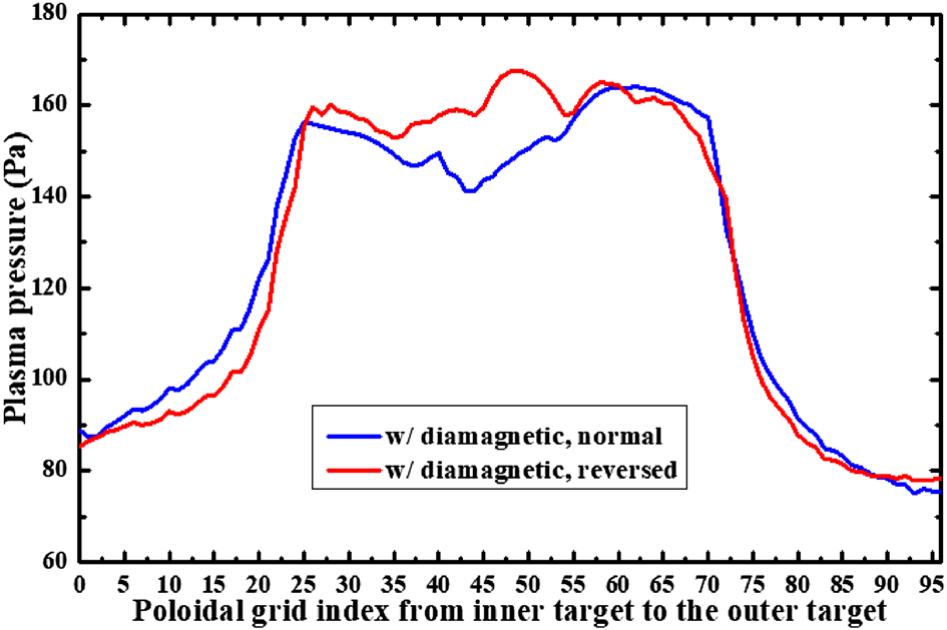
The plasma pressures at separatrix from the inner target to the outer target for the simulation cases with only diamagnetic drift under normal and reversed toroidal field directions.

Figure 4 shows the radial diamagnetic drift velocities at separatrix for the simulation cases with only diamagnetic drift under normal and reversed toroidal field directions. Note that the actual radial location (*R*) of the grid starting from the separatrix (*R*_sep_) is used to represent the radial location as shown in Fig. 2, while the grid index starting from the inner target to the outer target is used to represent the poloidal location as shown in Fig. 4. The positive radial and poloidal directions can be seen in Fig. 3a. The X coordinate at 0 is the inner divertor target and at 96 is the outer divertor target shown in Fig. 4. The poloidal grid index of interface is 24 and the poloidal grid index of interface is 72. Both of the interfaces pass through the X point. The positive direction of the diamagnetic drift velocity shown in Fig. 4 points towards the outer SOL boundary. As we can see, the diamagnetic drift velocity has positive values at interface while negative values at interface for the case with normal toroidal magnetic field, which is consistent with the diamagnetic flow pattern shown in Fig. 3a. The flow directions are reversed with the reverse of toroidal field direction as can be seen from the red curve compared to the blue curve in Fig. 4. The reason to the large velocity in the divertor region (the inner divertor region: X coordinates from 0 to 24, the outer divertor region: X coordiantes from 72 to 96) while small velocity in the upstream region (X coordinates from 24 to 72) is mainly due to the dependence of diamagnetic drift velocity on the pressure gradient. The pressure gradient in the poloidal direction is able to cause a cross-field radial diamagnetic drift flow. In the upstream region, the pressure gradient along the poloidal direction should be small, which means the cross-field transport induced by diamagnetic drift is weak, which may explain why the diamagnetic drift velocity is small in the upstream region as can be seen in Fig. 4. In the downstream of the divertor region, there is significant pressure loss in the poloidal direction which causes a large pressure gradient and a large cross-field diamagnetic drift flow in the divertor region. Figure 5 shows the plasma pressure at separatrix from the inner target to the outer target for the two cases mentioned above. As we can see, there are significant pressure losses from the poloidal location of X-point (X coordinate of 24/72) to the inner and outer divertor targets (43.1% and 39% for the case with normal toroidal magnetic field, 46.5% and 27.1% for the case with reversed toroidal magnetic field), which result in the large radial diamagnetic velocities there as shown in Fig. 4. Note that the magnitude of the radial diamagnetic drift velocity at the poloidal grid index of 72 is much larger than that at the poloidal grid index of 24 in the case with normal toroidal magnetic field, while no such difference is observed in the case with reversed magnetic field. This could be due to other factors like the plasma density and magnetic field strength as the diamagnetic drift velocity is also determined by these parameters. The distributions of these parameters vary significantly in the simulation domain and the plasma drifts have big impacts on them.

**Figure 6:**
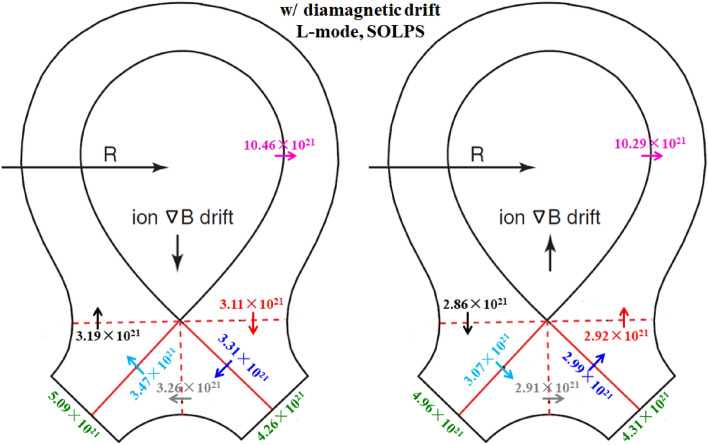
The numbers of total particles across interfaces to and the separatrix, along with those at the divertor targets for the two L-mode cases with diamagnetic drift by SOLPS.

**Figure 7:**
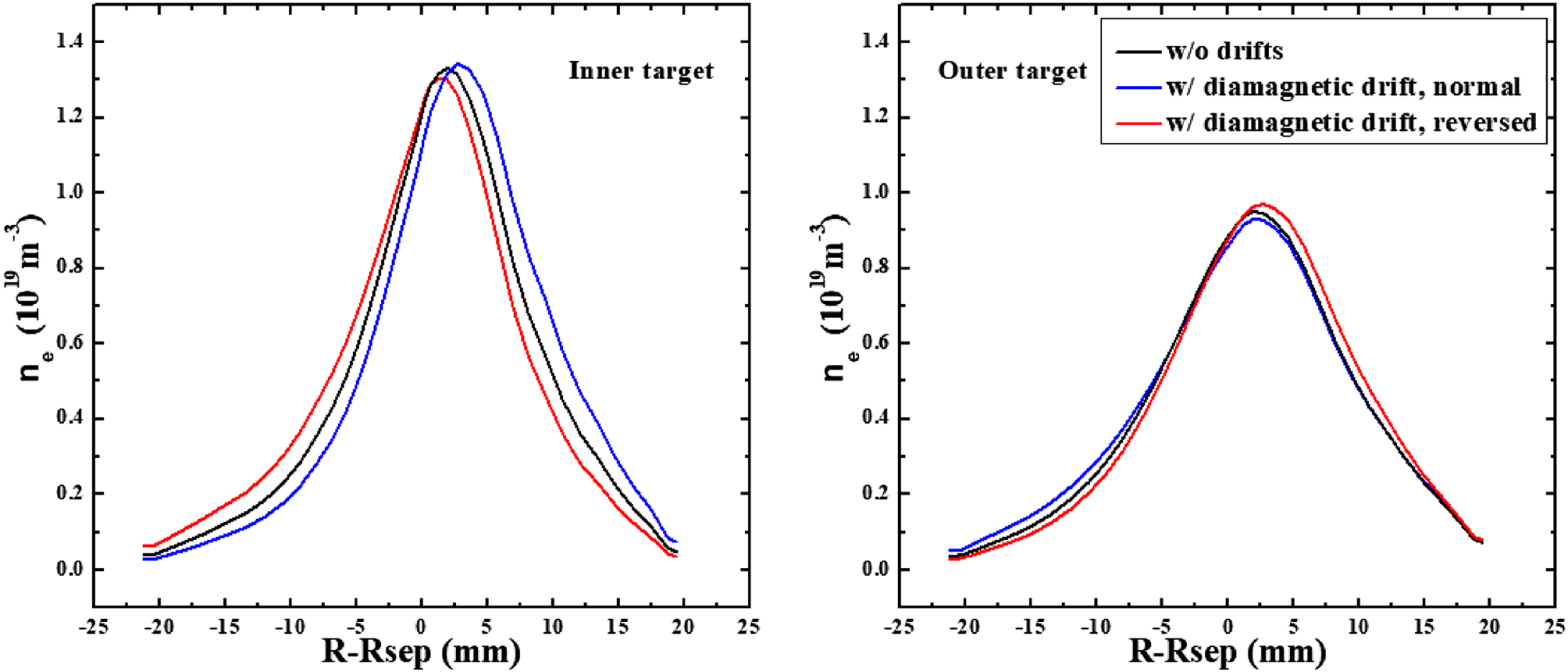
The plasma densities at the inner and outer targets for the simulation cases without drifts and with only diamagnetic drift.

To study the effect of diamagnetic drift on the particle flow pattern and in–out divertor asymmetry, the numbers of total particles across interfaces to are calculated and shown in Fig. 6. Note that the numbers of total particles mentioned here means the numbers of total particles per second and the interfaces to in the whole manuscript are the same as those shown in Fig. 3. Also shown are the numbers of total particles across separatrix from the core to the SOL and the numbers of total particles at the inner and outer divertor targets. The numbers of total particles across separatrix and at targets for the simulation case without drifts are shown in Table 1 to make comparisons. As we can see, the numbers of total particles across interfaces to are similar for both cases, indicating that the diamagnetic drift flows form a closed loop throughout the simulation domain. The numbers of total particles across separatrix and at inner and outer divertor targets for the two cases are also similar with the corresponding values from the case without drifts shown in Table 1, which mean almost no net particles are brought to the divertor target surfaces by the diamagnetic drift. The plasma densities at the inner and outer targets for the three cases are shown in Fig. 7. The distributions of plasma density are similar for the three cases and there is almost no change of in–out density asymmetry due to diamagnetic drift. Note that the divergence-free nature of the diamagnetic drift from previous theory is derived based on the property of the magnetic pre-sheath region. However, the results here show that even without solving the magnetic pre-sheath region, the diamagnetic drift flows caused by the radial pressure gradient and the poloidal pressure gradient are balanced by each other and the diamagnetic drift doesn’t change the particle balance between the inner and outer divertor regions. The simulation results indicate that the existence of the magnetic pre-sheath region may not be so important in determining the divergence-free nature of the diamagnetic drift as what the mathematical evaluation indicated. In general, the results here show that the diamagnetic drift wouldn’t cause a pronounced in–out density asymmetry, indicating that the in–out density asymmetry observed in experiment may be mainly caused by the E × B drift.

**Table 1:** The numbers of total particles across separatrix and at targets for the L-mode simulation case without drifts by SOLPS.

	Separatrix	Inner target	Outer target
w/o drifts	10.22 × 10^21^	4.93 × 10^21^	4.21 × 10^21^

The two components of the E × B drift, i.e., the radial and poloidal E × B drifts, operate dependently from each other. The radial E × B drift could drive the plasmas from one of the divertor leg region to the other, which is also the reason why Chankin et al. claim that the radial E × B drift is the main factor to the divertor density asymmetry^19^. The poloidal E × B drift however, could change the total distribution of poloidal particle flow and drive the particles into one side of the divertor region and out of the divertor region at the other side, which is also the reason why Rozhansky et al. claim that the poloidal E × B drift is the main factor to the divertor density asymmetry^18^. The drift directions are shown in Fig. 3b for tokamak plasmas with normal toroidal magnetic field.

**Figure 8:**
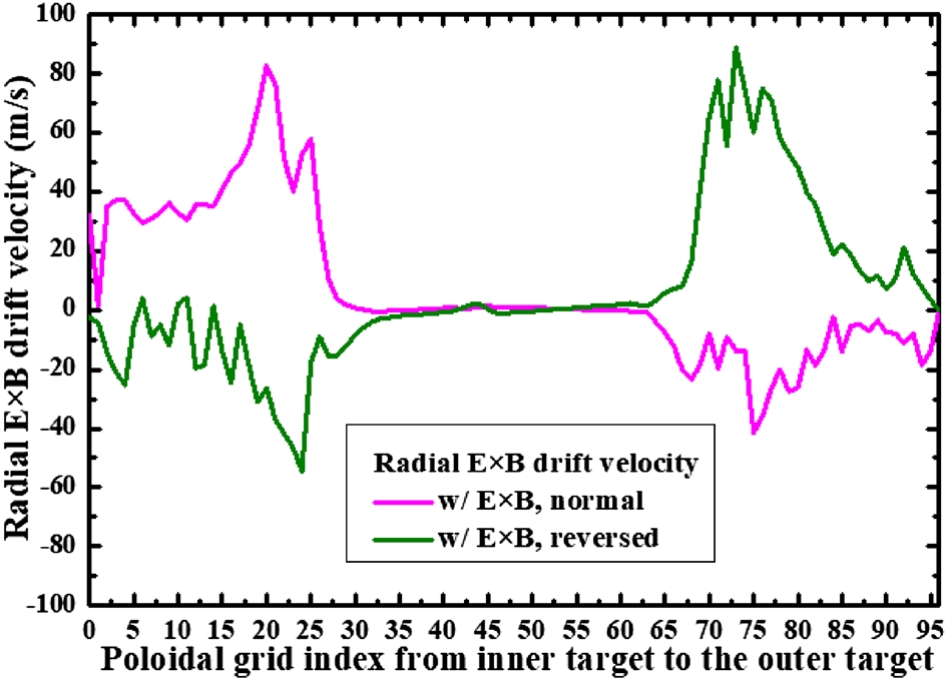
The radial E × B drift velocities at separatrix from the inner target to the outer target for the two cases with only E × B drift under normal and reversed toroidal magnetic fields.

**Figure 9:**
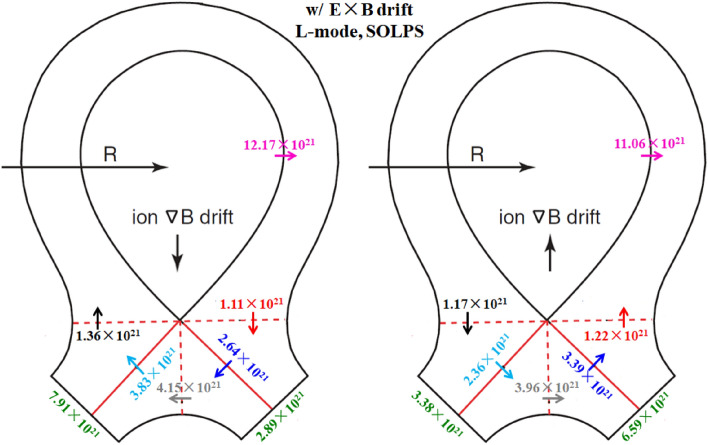
The numbers of total particles across interfaces to and the separatrix, along with those at the divertor targets for the two L-mode cases with E × B drift by SOLPS.

**Figure 10:**
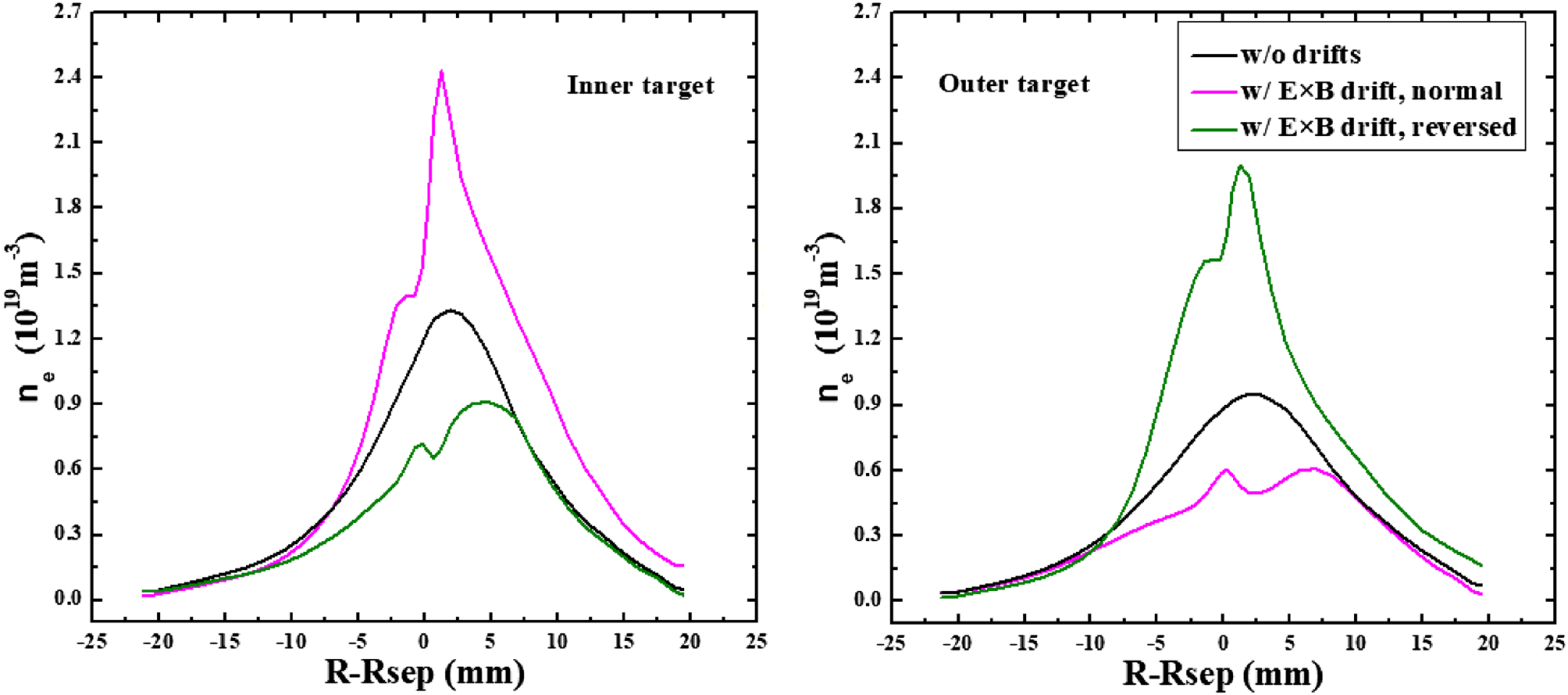
The distributions of plasma density at the inner and outer targets for the cases without drifts and with E × B drift under normal and reversed toroidal magnetic fields.

To study the effects of E × B drift on the particle flow pattern and in–out density asymmetry in the divertor region, the simulation cases with only E × B drift are run under both normal and reversed toroidal magnetic fields. Figure 8 shows the radial E × B drift velocities at separatrix from the inner target to the outer target for the two cases. As we can see, the radial drift velocity patterns for the two cases in Fig. 8 are very similar to those of Fig. 4, which seem reasonable since the E × B drift flows have similar paths with the diamagnetic drift flows as shown in Fig. 3. The magnitude of the radial E × B drift velocity at the divertor region (poloidal grid index in the ranges of 0–24 and 72–96) is much larger than that of the upstream region (poloidal grid index in the range of 24–72). This could be due to the larger poloidal electric field *E*_θ_ caused by the larger gradient of the electric field in the divertor region compared to the upstream region. However, the magnitudes of the velocities at interfaces and are different for each case. For the case with normal toroidal magnetic field, the absolute value of E × B drift velocity is larger at interface than interface as shown by the purple curve in Fig. 8. While it is opposite for the case with reversed toroidal magnetic field as shown by the green curve. The difference of the absolute values of E × B drift velocities at interfaces and may change the particle balance in the divertor region and eventually lead to the in–out asymmetry of density distributions. Figure 9 shows the numbers of total particles across interfaces to and the separatrix, along with those at the divertor targets. The numbers of particles across interfaces and are relatively small and almost equal for both cases, indicating that the E × B drift would not bring much net particles to the divertor region and the poloidal E × B drift in the SOL is not the main factor in inducing the in–out divertor asymmetry. The numbers of particles across interfaces to are much larger than those of interfaces and , resulting in a large amount of particles transported to one side of the divertor from the other side, which would break the particle balance between the inner and outer divertor region and cause a pronounced asymmetry of in–out divertor density asymmetry. There are strong asymmetries of total particles to the inner and outer divertor targets for the two cases compared to the case without drift shown in Table 1, which should be due to the strong particle transport between the two sides of the divertor region caused by the radial E × B drift. The particle flows would change directions with the reverse of toroidal magnetic field, which lead to the reversed in–out asymmetry of divertor particle flux for the two cases. Figure 10 shows the distributions of plasma density at the inner and outer targets for the cases without drifts and with E × B drift under normal and reversed toroidal magnetic fields. The plasma density at the inner target is much larger than that at the outer target for the case with normal toroidal magnetic field while the in–out asymmetry is reversed with the reverse of toroidal magnetic field, which are consistent with the in–out asymmetries of divertor particle flux for the two cases, showing that the radial E × B drift is the main factor in inducing divertor density asymmetry for the L-mode plasmas.

**Figure 11:**
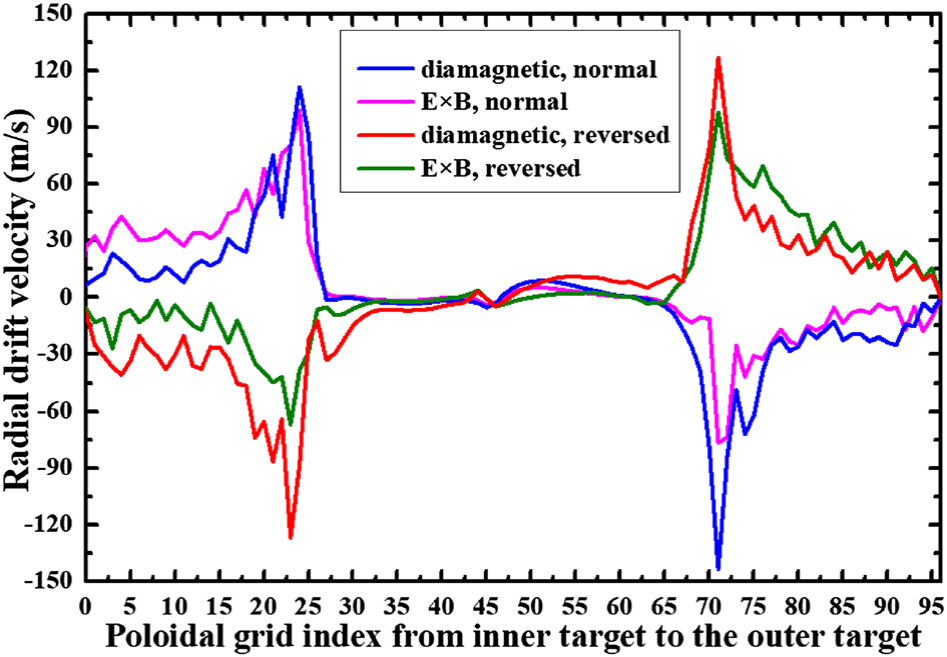
The radial E × B and diamagnetic drift velocities at separatrix from the inner target to the outer target for the two cases with both E × B and diamagnetic drifts under normal and reversed toroidal magnetic fields.

**Figure 12:**
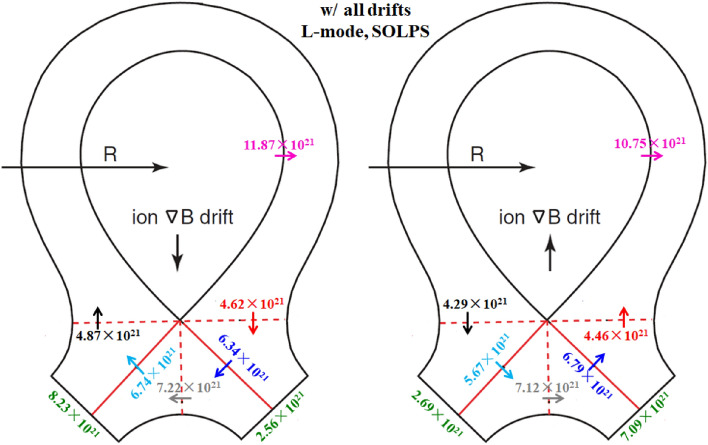
The numbers of total particles across interfaces to and the separatrix, along with those at the divertor targets for the two L-mode cases with all drifts by SOLPS.

**Figure 13:**
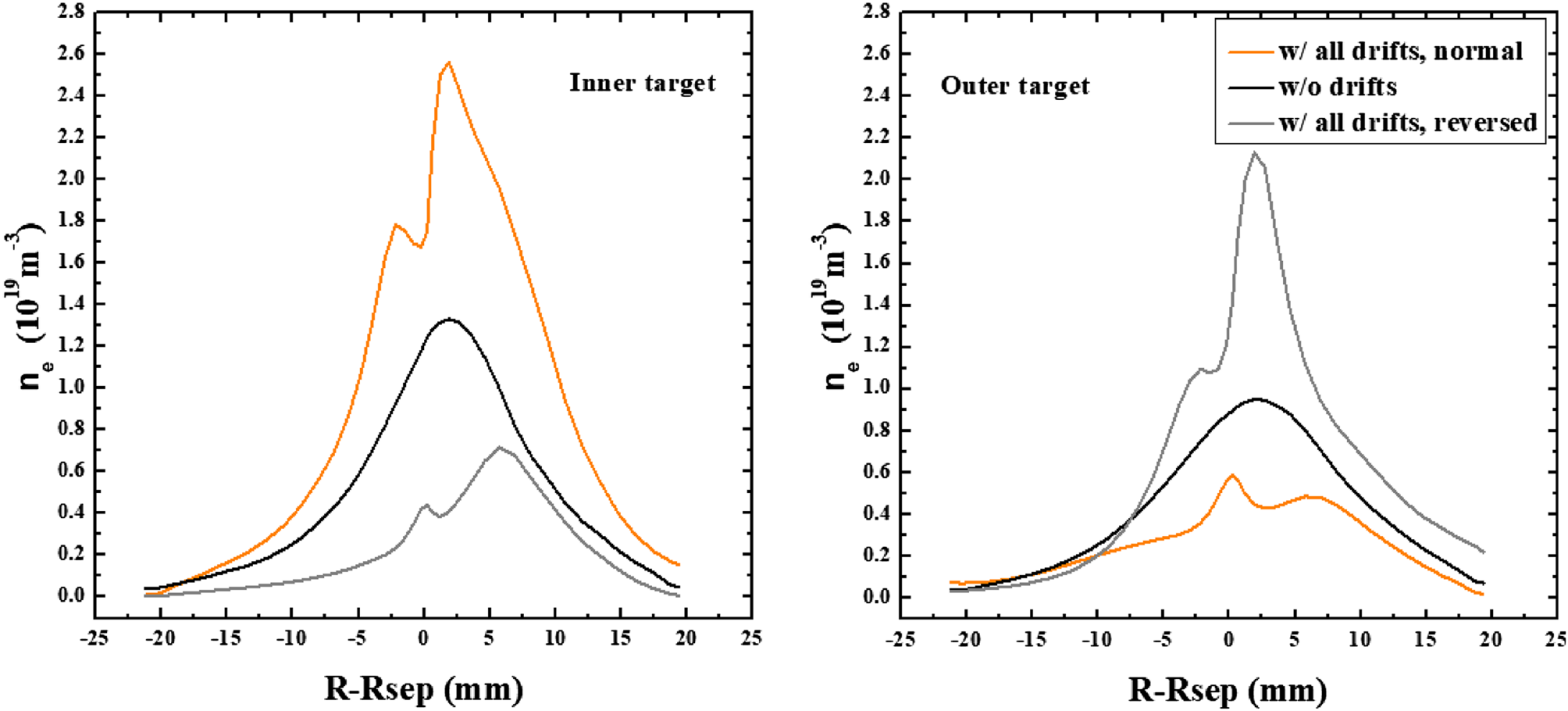
The distributions of plasma density at the inner and outer targets for the cases without drifts and with all drifts under normal and reversed toroidal magnetic fields.

The previous simulations already show that the E × B drift is responsible for the in–out density asymmetry while the diamagnetic drift doesn’t have much effect on it. However, these simulations are either with only diamagnetic drift or with only E × B drift. To study their integrated effects on the particle flow pattern and in–out density asymmetry in the divertor region, two cases are run with both drifts switched on under normal and reversed toroidal magnetic fields. Figure 11 shows the radial E × B and diamagnetic drift velocities at separatrix from the inner target to the outer target for these two cases. The E × B and diamagnetic drift velocities are similar with the corresponding velocities in previous simulation cases with only diamagnetic or E × B drift, indicating that the diamagnetic and E × B drift flows don’t have much effect on each other. The numbers of total particles across interfaces to for these two cases are shown in Fig. 12. As we can see, the numbers of particles across interfaces to are almost equal to the corresponding data in Fig. 6 and Fig. 9 combined. The numbers of particles across interfaces and are almost equal, indicating that drifts don’t have much effect on the number of total particles to the divertor region. The numbers of total particles across interfaces to are much larger than those of interfaces and , resulting in a large amount of particles been transported to one side of the divertor from the other side, which should be attributed by the radial E × B drift. The overall in–out asymmetries of divertor particle flux are similar with the data in Fig. 9, showing that the diamagnetic drift has almost no effect on the net particles to the divertor surface. Figure 13 shows the distributions of plasma density at the inner and outer targets for the cases without drifts and with all drifts under normal and reversed toroidal magnetic fields. There are pronounced in–out asymmetries for the two cases with all drifts and their asymmetries are opposite to each other. In general, the density in–out asymmetries in Fig. 13 are quite similar with the results in Fig. 10, which suggest that the radial E × B drift is the main factor to the divertor in–out asymmetries.

## Effects of drifts on the particle flow pattern and in–out asymmetry of plasma density in the divertor region for H-mode simulations with BOUT++ 

In the last section, the results from L-mode simulations by SOLPS are presented. This section will introduce the effects of drifts on the particle flow pattern and in–out asymmetry for the H-mode simulations by BOUT++ .

**Figure 14:**
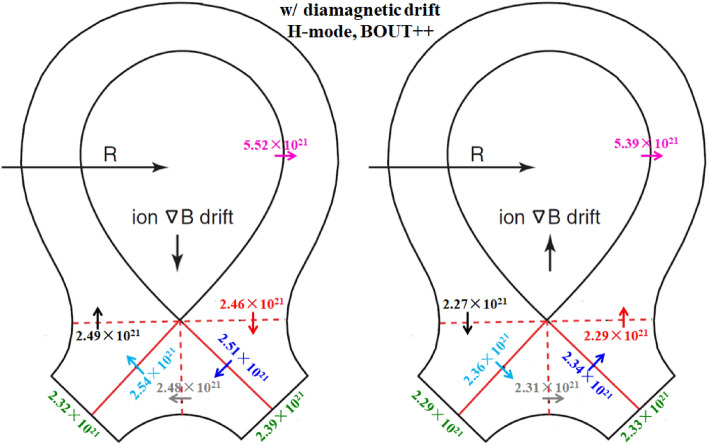
The numbers of total particles across interfaces to and the separatrix, along with those at the divertor targets for the two H-mode cases with diamagnetic drift by BOUT++ .

**Figure 15:**
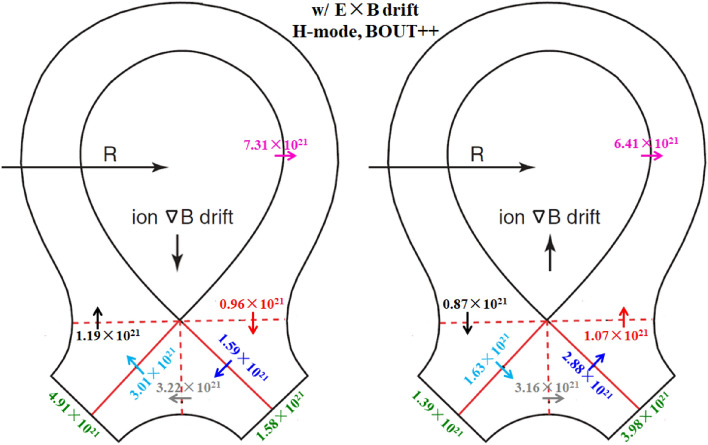
The numbers of total particles across interfaces to and the separatrix, along with those at the divertor targets for the two H-mode cases with E × B drift by BOUT++ .

**Figure 16:**
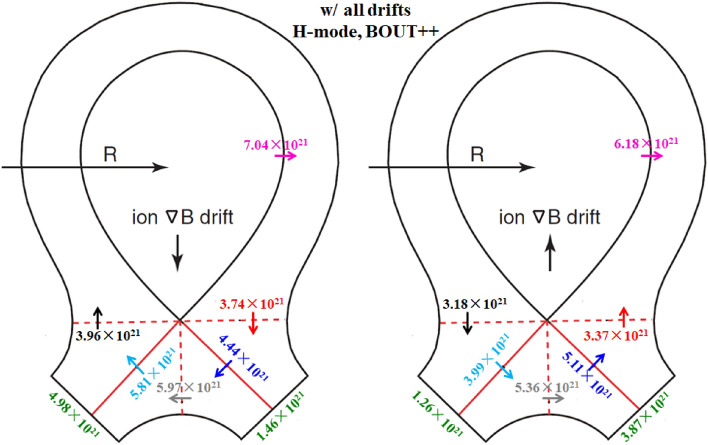
The numbers of total particles across interfaces to and the separatrix, along with those at the divertor targets for the two H-mode cases with all drifts by BOUT++ .

The simulation cases without drifts, with only diamagnetic drift, with only E × B drift and with all drifts under normal and reversed toroidal magnetic fields are run by the BOUT++ code. Table 2 shows the numbers of total particles across separatrix and at the inner and outer divertor targets for the simulation case without drifts. The number of particles at the inner target is almost the same as that of the outer target, which is a little different from the SOLPS results as the data in Table 1 shows that the number of particles at the inner target is a little more than that at the outer target. Figure 14 shows the numbers of total particles across interfaces to and the separatrix, along with those at the divertor targets for the two cases with diamagnetic drift. The numbers of particles that across to are similar and the numbers of particles at the inner and outer targets are almost the same, which is consistent with the results of L-mode simulation in Fig. 6, showing that the diamagnetic drift doesn’t change the in–out asymmetry of particle distributions. The results in Fig. 14 again have confirmed the divergence free nature of diamagnetic drift. Note that the numbers of particles across separatrix shown in Fig. 14 are only half of those in Fig. 6, which should be due to the transport barrier in the pedestal region for H-mode scenario. However, the numbers of particles across interface are about 79% of those in Fig. 6, which indicate that the diamagnetic drift flows are relatively stronger for H-mode plasmas than those of L-mode plasmas. There are many reasons that could lead to the big differences between the results of H-mode and L-mode cases mentioned above. The codes are different for the simulations of H-mode and L-mode plasmas. Previous results show that the differences of divertor particle decay widths from the two codes with similar simulation conditions could reach as much as 30%, while the differences of the numbers of total particles (the integrals of particle fluxes along the divertor targets) are much smaller than those of the particle flux decay widths. There are more important factors that could expand the gap between the diamagnetic flows of the H-mode and L-mode scenarios like the plasma density and the gradient of plasma pressure as the diamagnetic flows are determined by these parameters. The H-mode plasmas commonly have larger plasma densities and pressure gradients and could lead to the larger diamagnetic drift flows compared to the L-mode plasmas. Although it is hard to justify the big difference quantitatively since the two cases are different in many ways, it is qualitatively reasonable. The numbers of particles across interfaces to and the separatrix for the two cases with E × B drift are shown in Fig. 15. The E × B drift flows are also relatively stronger for the H-mode plasmas compared to the L-mode plasmas for similar reasons as the diamagnetic drift since the plasma densities and the gradients of electrostatic potentials (or equivalently, the *E*_r_/*E*_θ_) are usually larger in H-mode scenario compared to the L-mode scenario. The in–out asymmetries of divertor particle flux are significantly expanded by the E × B drift. Note that the in–out asymmetries of divertor particle flux are stronger in Fig. 15 than those of Fig. 9 as the ratios of inner to outer divertor particle fluxes are 3.11 and 0.35 for the two cases in Fig. 15 while the corresponding ratios are 2.74 and 0.51 for the data in Fig. 9, which should be due to the larger E × B drift effect. Figure 16 shows the numbers of particles across interfaces to and the separatrix for the two cases with all drifts. Compared with the results in Fig. 15, the inclusion of diamagnetic drift significantly increases the drift flows across the interfaces to . However, similar with the results in Fig. 12, it doesn’t change the in–out asymmetries of divertor particle fluxes due to its divergence free nature. In this section, the target density profiles are not given for simplicity since the results from H-mode simulations are similar with those of the L-mode simulations. In general, the results from both the L-mode and H-mode simulations by the two codes respectively have confirmed the divergence free nature of diamagnetic drift and the in–out divertor density asymmetries are mainly caused by the radial component of the E × B drift.

**Table 2:** The numbers of total particles across separatrix and at the inner and outer divertor targets for the H-mode simulation case without drifts by BOUT++ .

	Separatrix	Inner target	Outer target
w/o drifts	5.27 × 10^21^	2.26 × 10^21^	2.31 × 10^21^

## Summary and conclusions

A study of the effects of drifts on the particle flow pattern and in–out plasma density asymmetry in the divertor region for L-mode and H-mode plasmas is carried out for EAST discharges by the edge plasma transport codes SOLPS and BOUT++ . An EAST H-mode discharge #48,337 is chosen for the simulation. It is a LSN discharge with ion B × ▽B direction towards the X-point. The simulation of L-mode plasmas is done by SOLPS while the simulation of H-mode plasmas is done by BOUT++ . The radial transport coefficients for particles and energy are set to be 0.4 m^2^/s and 1.6 m^2^/s in the entire simulation domain for the L-mode plasma simulations, while the “U” shaped profiles of transport coefficients are set in the simulations for the H-mode plasmas. The toroidal magnetic field direction is artificially reversed in the codes to study the effects of different drift directions on the divertor particle flow pattern and the in–out asymmetry of divertor plasma density. The diamagnetic drift could induce a large particle flow throughout the entire divertor region. However, it seems to have no effect on the in–out asymmetry of divertor particle flux and plasma density due to its divergence-free nature. The E × B drift would not bring much net particle flux to the divertor region. However, the radial component of the E × B drift could lead to the transport of a large amount of particles from one side of the divertor to the other side, resulting in strong in–out asymmetries of divertor particle flux and density. The directions of the flows induced by diamagnetic and E × B drifts would be reversed with the reverse of toroidal magnetic field direction. The density in–out asymmetry caused by E × B drift is reversed with the reverse of E × B drift flow direction. The results from the simulations of H-mode plasmas with BOUT++ are similar to those of the L-mode plasmas with SOLPS except that the drift effects seem to be slightly larger in the H-mode plasmas compared to the L-mode plasmas. In general, the results from both the L-mode and H-mode plasma simulations show that the poloidal E × B drift doesn’t change the particle balance in the whole divertor region. The radial E × B drift however, drives a large amount of particles from one of the divertor leg region to the other, which may have caused the divertor in–out asymmetry of plasma density. So our results for both L-mode and H-mode scenarios are in favor of Chankin’s claim, i.e., the radial E × B drift plays a more important role in determining the divertor density in–out asymmetry.

## Data Availability

The datasets and analyses details are available from the corresponding author on reasonable request.
